# PrepCare: a two-stage framework for infectious disease prioritization and alerts for nursing preparedness

**DOI:** 10.3389/fpubh.2026.1776109

**Published:** 2026-03-27

**Authors:** Jing Zhu, Longsheng Xie, Chengguo Zhuo, Chunlan Yu, Wenying He

**Affiliations:** The Affiliated Hospital of Southwest Medical University, Luzhou, Sichuan, China

**Keywords:** infectious disease surveillance, LightGBM regressor, nursing resource planning, STL decomposition, two-stage framework

## Abstract

**Introduction:**

Nursing services often face resource constraints, while reliable disease specific prioritization signals are difficult to obtain from limited routine data. We therefore developed PrepCare, a two-stage interpretable framework for infectious disease prioritization and alerting to support nursing resource planning.

**Methods:**

PrepCare combines data-adaptive disease ranking with Seasonal-Trend decomposition using LOESS (STL)-based alerting. Diseases were prioritized using a multi-indicator composite index based on six pillars: burden, recent incidence, risk, trend, policy-informed severity, and a burden-severity interaction term. Pillar weights were assigned using an information entropy principle, and a Light Gradient Boosting Machine (LightGBM) regressor was used to capture non-linear interactions. SHapley Additive exPlanations for Tree-based models (TreeSHAP) were used to obtain a system-wide importance ranking. The top 15 diseases were then selected for alert assessment. STL was applied to log counts to remove seasonality and trend, and anomalies were detected from the residuals using calibrated thresholds.

**Results:**

The framework produced a ranked list of priority diseases and generated alert signals for the top 15 diseases. STL-based alerting was supported by strong annual seasonal components, a reduction in residual variance, and decreased lag-1 autocorrelation after decomposition, indicating improved separation of recurrent patterns from irregular deviations.

**Discussion:**

PrepCare offers a reproducible, interpretable, and label-agnostic “rank-selectalert” workflow for infectious disease early warning. The framework may support nursing staff scheduling, stock preparation, and risk communication, thereby enhancing preparedness in resource-constrained settings.

## 1 Introduction

Infectious diseases continue to pose persistent and uneven threats to health systems (1). High-risk pathogens such as human immunodeficiency virus (HIV) (2), tuberculosis (3), influenza (4), respiratory syncytial virus (5), and emerging coronaviruses spread through communities with variable speed and intensity, and their surges often materialize in hospital wards before hospitals can adapt (6, 7). These events translate into increased nursing workload, pressure on bed capacity, supply consumption, and the need for rapid patient education, triage, and isolation practices. When acceleration in admissions or case notifications is detected late, staff redeployment and supply chain actions become reactive rather than preventive, thereby increasing costs and amplifying inequities in access to timely care (8).

Nursing teams manage many of the most immediate downstream effects of infectious surges, such as short-cycle rota updates, bedside skill-mix adjustments, isolation and cohorting workflows, and quick stock planning for protective equipment and essential consumables (9, 10). A surveillance method that is interpretable at the disease level and updated from routine monthly reports is therefore directly relevant to nursing preparedness and operational planning (11, 12). Timely detection of diseases with increasing near-term incidence risk is therefore a practical priority for nursing leadership and infection prevention teams (11, 13, 14). Beyond recognizing which conditions are trending upward, clinical operations benefit from risk-informed prioritization and month-stamped anomaly alerts. These signals support proactive scheduling, unit-level skill-mix planning, allocation of personal protective equipment and key medications, and tailored communication with patients and families (10, 15). In community settings, the same operational logic can guide outreach timing, vaccine delivery logistics, and coordination with long-term care and primary care partners (16–18).

Yet many surveillance workflows remain retrospective and descriptive (19–21). In many settings, routine practices still depend on retrospective bulletins, manual monitoring, and simple fixed-threshold rules on raw counts, which can miss contextual seasonality and disease-specific volatility (13, 22). Tables of monthly totals summarize what has happened but rarely indicate what may happen next or with what confidence (11). In syndromic surveillance settings, threshold-based alerts are sensitive to noise and seasonality and may trigger either too late or too often (23–25). Hence, nursing teams require a method that is auditable, low-overhead, and capable of converting routine surveillance data into actionable guidance for the next planning cycle (10).

In this study, we propose a two-stage workflow that (i) prioritizes diseases with the highest near-term concern by computing a composite score from multiple surveillance indicators, and (ii) concentrates monitoring on the top-ranked diseases and issues alerts for atypical increases using thresholded residuals from Seasonal-Trend decomposition using LOESS (STL) (26). We focus on nurses because they are the first operational layer responsible for translating surveillance signals into staffing, stock, and workflow adjustments under tight resource constraints. This motivates low-overhead and interpretable signals aligned with routine monthly operational planning. Our empirical study uses national-level monthly notifications from the Chinese NDCPA (September 2022–August 2025). However, the pipeline itself is data-source agnostic and can be adapted to other settings with comparable disease-by-time count data and locally defined severity priors.

This study makes three key contributions. First, it introduces a multi-indicator prioritization pipeline that combines entropy-based weighting with a teacher-student ranking framework, enabling effective disease prioritization under weak or label-sparse settings. Second, it maintains transparency by using SHapley Additive exPlanations for Tree-based model (TreeSHAP) to quantify how each indicator contributes to the ranking, providing actionable explanations for decision makers. Third, it couples prioritization with STL residual-threshold alerting to detect unusual increases in near real time, offering an interpretable early-warning mechanism that supports nursing workforce and resource preparedness.

## 2 Methods

### 2.1 Data collection

We utilized monthly case counts publicly released by the National Disease Control and Prevention Administration of China (NDCPA) (27). The dataset comprises 47 nationally notifiable infectious diseases, encompassing statutory Classes A, B, and C. We extracted a 36-month window (September 2022 to August 2025) to balance capturing recurrent seasonality with responsiveness to recent changes. These monthly counts serve as the sole input for both the multi-indicator ranking and the STL-based alerting methods described in subsequent sections.

### 2.2 Multi-indicator ranking

**Figure 1:**
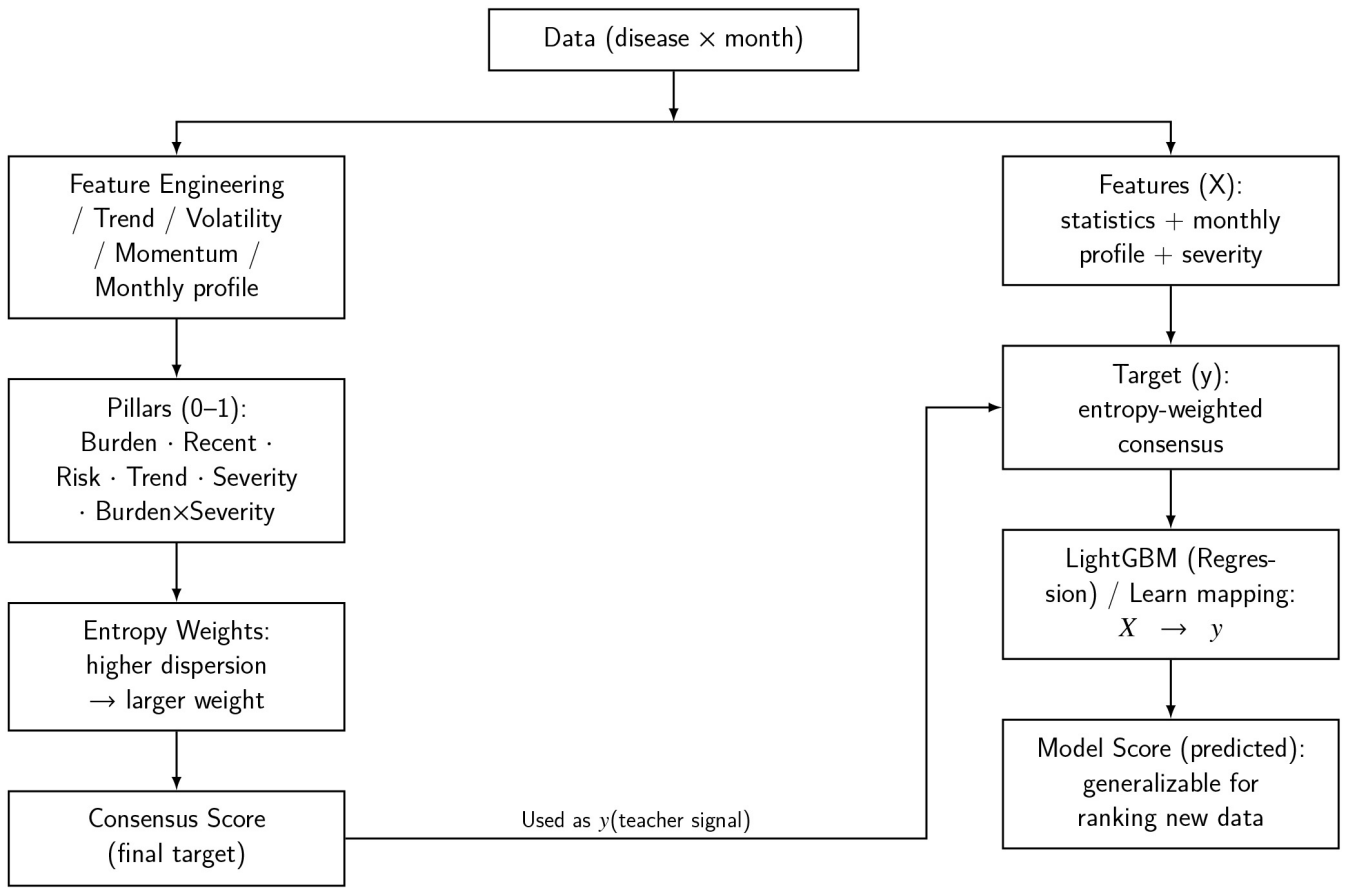
Overview of the two-path, teacher-student ranking pipeline. **Left**: six interpretable pillars (Burden, Recent, Risk, Trend, Severity, and Burden × Severity) are constructed from routine monthly counts, normalized and robustly processed, and combined via entropy weighting into a single consensus target *y*. **Right**: richer features *X* (summary statistics, normalized monthly profile, and the severity prior) are used to train a gradient-boosted tree regressor (LightGBM) *f*(*X*), producing a reusable model score for ranking that can be refreshed as new monthly data become available.

As shown in Figure 1, the ranking framework follows a two-path teacher-student design that turns disease-by-month series into portable and interpretable scores. On the left path, six interpretable pillars: Burden, Recent, Risk, Trend, Severity, and the interaction Burden × Severity are derived under a common scaling and robust treatment. Information-entropy weighting across six pillars, using cross-disease dispersion to set data-adaptive weights, produces a single consensus target *y* for nursing-relevant importance.

On the right path, a richer feature set *X* (statistics, the normalized monthly profile, and the severity prior) trains a Light Gradient Boosting Machine (LightGBM) model (28, 29) to approximate *y* and output a model score ŷ that can be updated as new monthly data arrive. To avoid target leakage, the interaction pillar is used only in constructing *y* and is not fed as an input feature. Exact additive attributions from TreeSHAP (30, 31) ensure that the scorer remains transparent while retaining generalisability for operational use. The teacher target thus ensures relevance to the nursing domain, and the student model ensures robust temporal portability for actionable nursing planning decisions.

#### 2.2.1 Entropy-based pillar weighting and consensus objective

We compressed nursing-relevant importance into a single objective by aggregating six interpretable pillars derived from routine disease-by-month counts. Monthly observations *x*_*t*_ are stabilized via log(1+*x*_*t*_) to mitigate heavy tails and scale heterogeneity; missing and extreme values are handled with standard imputation and winsorisation (5th-95th percentiles) (32). All pillar components are then mapped to [0, 1] to ensure comparability across diseases.

Pillar definitions are as follows:

Burden (cases): overall workload, quantified by log(1+total_cases).

Recent (recency): short-window mean activity (e.g., last 6 months), optionally computed as log(1+recent_avg).

Risk (volatility/extremes): dispersion and tail behavior captured by a convex combination of log-scale variability and the maximum standardized deviation (33):


$$\begin{array}{l} \text{Risk}=0.6\text{st}{\text{d}}_{\log\left(1+x\right)}+0.4\underset{t}{\max}\left(\frac{x_{t}-\bar{x}}{s}\right), \end{array}$$ (1)

where {*x*_*t*_} is the monthly series (untransformed), and $\bar{x}$ and *s* denote its mean and standard deviation, respectively. Here, Risk is the empirical volatility/extremes index rather than the probability of occurrence. Severity priors reflect consequence (clinical/public-health impact), while Risk captures temporal instability; they are complementary and need not rank diseases identically.

Trend: least-squares slope of log(1+*x*_*t*_) over time, winsorised at the 5th-95th percentiles for robustness.

Severity prior: a policy and clinical mapping (e.g., statutory category) linearly rescaled to [0, 1]. Diseases classified as Class A and B carry a relatively higher severity score (27). We have included the severity scores we assigned based on disease severity in the Supplementary Material Section 1.

Burden × Severity: an interaction pillar emphasizing scenarios that are simultaneously high-volume and high-acuity.

The rationale for selecting these six pillars is as follows. The set was chosen to be actionable for nursing operations, identifiable from routine surveillance, and mutually complementary. Burden links directly to expected workload in beds, personal protective equipment, and staff time. Recent activity emphasizes near-term pressure that aligns with rota planning and re-stocking cycles. Risk captures volatility and extreme behavior, reflecting surge potential and uncertainty that warrant buffer capacity even when the mean level is moderate, and is operationalized in Equation 1. Trend encodes directionality (escalation vs. remission), enabling proactive allocation rather than reactive response. A severity prior aligns prioritization with clinical and policy acuity that raw case counts may under-represent, including isolation needs and transmission-control requirements. Finally, the interaction between burden and severity isolates conditions that are simultaneously high-volume and high-acuity and therefore most consequential for preparedness. Together, these pillars span volume, recency, variability, trajectory, policy-informed gravity, and their key interaction with minimal redundancy. As a concrete example, a disease with high burden but stable month-to-month counts can have high Burden but moderate Risk, whereas a disease with lower mean burden but pronounced spikes can show higher Risk and therefore be flagged for surge-awareness.

To assign data-adaptive weights without manual tuning, we used information entropy across diseases (34). Let *Z*_*ij*_≥0 be disease *i*'s score on pillar *j* (*j* = 1, …, 6) after common scaling, and let *n* be the number of diseases. The across-disease proportions and pillar entropy values used in the weighting scheme are defined in Equations 2, 3. Define the across-disease proportions


$$\begin{array}{l} p_{ij}=\frac{Z_{ij}}{\sum_{i=1}^{n}Z_{ij}}. \end{array}$$ (2)

The entropy of pillar *j* is


$$\begin{array}{l} e_{j}=-\frac{1}{\ln\;n}\overset{n}{\underset{i=1}{\sum }}p_{ij}\ln\;p_{ij}. \end{array}$$ (3)

Weights favor pillars with greater cross-disease dispersion:


$$\begin{array}{l} w_{j}=\frac{1-e_{j}}{\sum_{\ell =1}^{6}\left(1-e_{\ell }\right)}. \end{array}$$ (4)

The consensus objective (teacher target) for disease *i* is


$$\begin{array}{l} y_{i}=\overset{6}{\underset{j=1}{\sum }}w_{j}{\tilde{Z}}_{ij}, \end{array}$$ (5)

where ${\tilde{Z}}_{ij}$ denotes the normalized pillar matrix. Pillars with higher cross-disease dispersion receive larger *w*_*j*_, whereas uniformly uninformative pillars are down-weighted. To prevent target leakage, the interaction pillar (Burden × Severity) is used solely in constructing *y* and is not supplied as an input feature; the predictive model is allowed to recover interactions from primitive covariates when supported by the data.

#### 2.2.2 Learning and prediction

The goal of this component is to develop a portable scorer that maps routinely derived features to a scalar score, thereby enabling a cross-disease ranking that can be applied prospectively to new months. For each disease *i*, we constructed a feature vector *X*_*i*_ from the same surveillance series used to form the consensus target, but with richer granularity. Specifically, *X*_*i*_ includes (i) distributional and stability summaries of the log-transformed counts (e.g., log(1+total), recent–baseline contrasts, variance, coefficient of variation, extrema, and the least-squares slope on log(1+*x*_*t*_) with 5th–95th percentile winsorisation), (ii) the normalized monthly profile (a 12-element composition describing within-year allocation and seasonal shape), and (iii) the policy-informed severity prior as a static covariate. Consequently, the dimensionality of *X* is substantially larger than the six-pillar set (*p*≫6), allowing the learner to capture non-linearities and interactions that a fixed linear mixture of pillars cannot. The interaction pillar *Burden*×*Severity* is intentionally excluded from *X* to avoid target leakage. The learner is allowed to recover interactions from primitive covariates where supported by the data.

A gradient-boosted decision-tree regressor (LightGBM) model is fitted to approximate the entropy-weighted consensus target *y* obtained in Section 2.2.1. Let $X_{i}\in {\mathbb{R}}^{p}$ denote the feature vector for disease *i* (constructed from routinely derived summaries and monthly profiles; *p* is the number of features). Fitting LightGBM yields a function *f*:ℝ^*p*^ → ℝ that maps these features to a scalar importance score. The resulting model score is thus defined by Equation 6, so that the model's output for disease *i* is


$$\begin{array}{l} {ŷ}_{i}=f\left(X_{i}\right). \end{array}$$ (6)

We sorted {ŷ_*i*_} in descending order to obtain the final ranking used for decision-making. When ties occur, they are resolved using dense ranking to maintain the stability of the effective top-K set for downstream analyses (35). To avoid ad hoc tuning, hyperparameters were selected from a prespecified candidate set using leave-one-disease-out (LODO) (36, 37) cross-validation and a deterministic selection rule. The primary intent is to develop a stable scorer that generalizes well under consistent feature engineering, rather than optimizing for in-sample fit.

Learning *f*(·) rather than ranking directly by the consensus target offers two advantages that are operationally relevant for nursing preparedness. First, expressivity where the feature set retains fine-scale distributional information and temporal shape, enabling *f*(·) to capture non-linearities and interactions that a fixed linear aggregation of six pillars cannot represent. Second, temporal portability in which once *f*(·) is trained, the identical feature construction can be applied to prospectively arriving months, yielding comparable ŷ without recomputing entropy weights at each update. To preserve a clean separation between target construction and prediction, all transformations that define *X* are specified independently of the fitted weights and applied identically at deployment.

The resulting score ŷ is used to identify a tractable subset of diseases for intensified monitoring and alert evaluation.

#### 2.2.3 Model training and validation

The ranking learner is implemented with the Python LightGBM package (version 4.6.0), using a regression objective to fit the entropy-weighted consensus target *y* rather than directly forecasting monthly case counts. The training cohort contains 47 disease-level samples, and each sample is represented by the full feature vector *X*_*i*_ described above. To improve reproducibility and avoid ad hoc tuning with this small sample size, we compared prespecified candidate configurations spanning the main LightGBM control dimensions (tree complexity, learning rate, ensemble size, feature subsampling, and minimum leaf sample size), with a fixed random seed to ensure deterministic runs. We intentionally used a small candidate set rather than a full Cartesian grid to limit tuning variance and selection bias. Candidates were evaluated using disease-level LODO cross-validation and selected using a deterministic rule. The hyperparameter candidates and selection criteria are reported in Supplementary Tables S2, S3.

In fold *i*, disease *i* is held out as test data, and the model is trained on the remaining 46 diseases; the corresponding LODO prediction rule is given in Equation 7:


$$\begin{array}{l} {ŷ}_{i}^{\left(-i\right)}=f^{\left(-i\right)}\left(X_{i}\right),\;i=1,\ldots ,N,N=47, \end{array}$$ (7)

where *f*^(−*i*)^ denotes the model fitted without disease *i*, and ${ŷ}_{i}^{\left(-i\right)}$ is the corresponding out-of-fold (OOF) prediction. This split design aligns with the analytical unit of the ranking task and avoids leakage between the train and test folds.

Validation performance is reported using five complementary metrics that jointly evaluate score accuracy and ranking consistency. The five validation metrics used for this assessment are formally defined in Equations 8-12. Let $e_{i}={ŷ}_{i}^{\left(-i\right)}-y_{i}$ denote out-of-fold errors, and let ŷ^OOF^ collect all ${ŷ}_{i}^{\left(-i\right)}$. For average absolute score error, we use MAE (38, 39) as:


$$\begin{array}{l} \text{MAE}=\frac{1}{N}\overset{N}{\underset{i=1}{\sum }}|e_{i}|, \end{array}$$ (8)

where *N* = 47 is the number of diseases in LODO evaluation, and |*e*_*i*_| is the absolute out-of-fold error for disease *i*. To place stronger weight on larger errors, we used RMSE (39) as:


$$\begin{array}{l} \text{RMSE}=\sqrt{\frac{1}{N}\overset{N}{\underset{i=1}{\sum }}e_{i}^{2}}, \end{array}$$ (9)

where $e_{i}^{2}$ penalizes larger deviations more strongly than MAE. For global rank-order consistency between teacher and predicted scores, we used Spearman correlation (40) as:


$$\begin{array}{l} \rho_{s}=\text{Corr}\left(\text{rank}\left(y\right),\text{rank}\left({ŷ}^{\text{OOF}}\right)\right), \end{array}$$ (10)

where rank(·) is the rank transform and Corr(·, ·) is Pearson correlation on the ranked vectors. For pairwise rank-order consistency, we used Kendall's τ (41) as:


$$\begin{array}{l} \tau_{k}=\text{KendallTau}\left(\text{rank}\left(y\right),\text{rank}\left({ŷ}^{\text{OOF}}\right)\right). \end{array}$$ (11)

where KendallTau(·, ·) measures concordance across all disease pairs. For top-list consistency, we defined the top-15 overlap rate (42) as:


$$\begin{array}{l} \text{Overla}{\text{p}}_{15}=\frac{\left|T_{15}\cap P_{15}\right|}{15}, \end{array}$$ (12)

where $T_{15}$ and $P_{15}$ denote the teacher and LODO-predicted top-15 disease sets, respectively, and $\left|T_{15}\cap P_{15}\right|$ is the number of diseases shared by both shortlists. All fold-level out-of-fold predictions and summary metrics are exported along with the model artifacts to ensure precise reproducibility of the training-validation workflow. The full tested candidate list and candidate-wise LODO performance are reported in Supplementary Material Section 2.

#### 2.2.4 Interpretability of the ranking model

We used TreeSHAP to obtain exact additive attributions for the tree-ensemble scorer, providing transparent explanations without retraining or approximation (30, 31). The additive TreeSHAP decomposition of the model score is shown in Equation 13. For each disease *i* with feature vector *X*_*i*_, the model score decomposes as


$$\begin{array}{l} {ŷ}_{i}=\phi_{0}+\overset{p}{\underset{k=1}{\sum }}\phi_{ik}, \end{array}$$ (13)

where ϕ_0_ is the model baseline and ϕ_*ik*_ is the contribution of feature *k* to sample *i*. This supports local explanations (why a specific disease scores high or low) and global summaries by aggregating |ϕ_*ik*_| across diseases to identify the dominant drivers of ranking. Because the attributions are defined on the input features, they can be grouped back to thematic blocks (e.g., burden-, trend-, or risk-related features) to align with pillar-level narratives used in operations. TreeSHAP's algorithmic form scales efficiently with tree depth and node count, making it practical for repeated re-scoring as new months arrive, while preserving a clean separation between target construction and prediction.

### 2.3 STL diagnostics

Based on the prioritization results from Section 2.2.2, we used STL as the preprocessing step to generate alerts for the top-ranked diseases. Our objective is to generate timely anomaly warnings for the next scheduling and stock-preparation cycle by extracting de-seasonalized, low-autocorrelation residuals suitable for simple thresholding while retaining an interpretable seasonal pattern for planning baselines. This alerting layer is not designed to predict the exact future peak month.

#### 2.3.1 STL decomposition

STL separates a series into trend, seasonal, and remainder (residual) components using local polynomial smoothing. The log-scale transformation and additive STL decomposition used here are defined in Equations 14, 15. Let *x*_*t*_ be monthly counts. We stabilize variance via


$$\begin{array}{l} y_{t}=\log\left(1+x_{t}\right), \end{array}$$ (14)

and assume an additive decomposition on the log scale


$$\begin{array}{l} y_{t}=T_{t}+S_{t}+R_{t}, \end{array}$$ (15)

where *T*_*t*_ is a slowly varying level, *S*_*t*_ is a seasonal component with period *m* (determined from data diagnostics), and *R*_*t*_ is the remainder. STL estimates (*T*_*t*_, *S*_*t*_) by alternating two Loess smoothers:

(i) Seasonal smoother: Split the series into *m* subseries by calendar position; locally smooth each subseries with a seasonal window and interpolate back to obtain a preliminary ${\hat{S}}_{t}$;
(ii) Trend smoother: Locally smooth the de-seasonalization series $y_{t}-{\hat{S}}_{t}$ with a (typically wider) trend window to obtain ${\hat{T}}_{t}$.

These steps are iterated, optionally with robust reweighting between iterations to downweight outliers and level shifts. The fitted components are denoted by ${\hat{T}}_{t}$ and ${\hat{S}}_{t}$, and the residual used for alerting is


$$\begin{array}{l} {\hat{R}}_{t}=y_{t}-{\hat{T}}_{t}-{\hat{S}}_{t}. \end{array}$$ (16)

To assess the suitability of STL for infectious disease case alerting, we demonstrate its effectiveness for alerting from four perspectives: seasonal significance, variance reduction, correlation attenuation, and annual cycle validation. Suitability is assessed on a calibration window of *L* months (we use *L* = 24) to avoid look-ahead.

Seasonal strength (43) is defined as


$$\begin{array}{l} S_{\text{strength}}=1-\frac{\text{Var}\left({\hat{R}}_{t}\right)}{\text{Var}\left({\hat{S}}_{t}+{\hat{R}}_{t}\right)}. \end{array}$$ (17)

It measures how strongly the seasonal component contributes to the original signal. Higher values indicate a stronger recurring seasonal pattern, supporting the use of de-seasonalization.

The variance ratio after de-seasonalization (44) is


$$\begin{array}{l} \text{VR}=\frac{\text{Var}\left({\hat{R}}_{t}\right)}{\text{Var}\left(y_{t}\right)}. \end{array}$$ (18)

This measures the variability that remains after removing trend and seasonality. Smaller values indicate cleaner residuals, which are better suited for threshold-based alerts.

The lag-1 autocorrelation (45) of residuals is


$$\begin{array}{l} \rho_{1}\left(\hat{R}\right)=\text{Corr}\left({\hat{R}}_{t},{\hat{R}}_{t-1}\right). \end{array}$$ (19)

This checks for remaining serial dependence; lower autocorrelation indicates that the residuals are closer to white noise, making quantile-calibrated exceedance rules more reliable.

To select the seasonal period, we examined the spectrum of *y*_*t*_. With $Y\left(\omega_{k}\right)=\overset{n}{\underset{t=1}{\sum }}y_{t}e^{-i\omega_{k}t}$ and ω_*k*_ = 2π*k*/*n* (46), the periodogram is


$$\begin{array}{l} I\left(\omega_{k}\right)=\frac{1}{n}{\left|Y\left(\omega_{k}\right)\right|}^{2}. \end{array}$$ (20)

A pronounced peak near ω ≈ 2π/12 indicates an annual cycle. Combined with high seasonal strength and improved residual diagnostics (low variance ratio and low ρ_1_), this motivates choosing *m* = 12 for the decomposition.

#### 2.3.2 Using STL for alerting

Alerts are raised on calibrated residuals so that exceedances reflect atypical deviations rather than seasonal peaks. The one-sided residual alert score and its quantile-calibrated threshold are defined in Equations 21, 22.

Using Equation 16, we define a one-sided, robust residual score on the calibration window C (first *L* months) as


$$\begin{array}{l} z_{t}=\frac{\max\left\{0,{\hat{R}}_{t}\right\}}{\text{MAD}\left(\left\{{\hat{R}}_{u}:u\in C\right\}\right)}, \end{array}$$ (21)

where $\text{MAD}\left(\left\{{\hat{R}}_{u}:u\in C\right\}\right)$ is the median absolute deviation of residuals over C, *z*_*t*_ is the MAD-scaled positive residual at month *t*. A per-disease threshold is set by quantile calibration (47) on C, and τ(ρ) is the disease-specific alert threshold obtained by quantile calibration with a minimum score floor τ_min_


$$\begin{array}{l} \tau \left(\rho \right)=\max\left\{{\mathrm{Quantile}}_{1-\rho }\left(\left\{z_{u}:u\in C\right\}\right),\tau_{\min}\right\}, \end{array}$$ (22)

the operator Quantile_1−ρ_(·) returns the (1−ρ) quantile (upper-tail cutoff) of the calibration scores $\left\{z_{u}:u\in C\right\}$. Here, ρ ∈ (0, 1) denotes the target exceedance rate in the calibration window. Operationally, ρ and τ_min_ play distinct roles: increasing ρ lowers the calibrated threshold and increases alert sensitivity (and alert burden), whereas τ_min_ prevents trivially small score fluctuations from triggering alerts. For operating-point selection, we evaluated a prespecified sweep ρ ∈ {0.01, 0.02, 0.05, 0.10, 0.20, 0.30} under the same calibration split and examined the burden—sensitivity trade-off. An alert is issued in monitoring months when *z*_*t*_ ≥ τ(ρ). In practice, alerts are generated after the official report for month *t* becomes available; we computed *z*_*t*_ using the observed value for month *t* and flagged whether that month is anomalous. Thus, the Stage-2 module performs contemporaneous anomaly flagging rather than exact forecasting of a future peak month.

Because ${\hat{S}}_{t}$ provides an explicit calendar-month baseline and ${\hat{T}}_{t}$ captures slow changes, alerts correspond to departures from expected seasonal and trend levels. Diagnostics Equations 17–19 justify this procedure: higher seasonal strength and lower variance ratio indicate effective extraction of the periodic component, whereas reduced autocorrelation yields residuals closer to white noise, improving the reliability of per-disease, quantile-calibrated exceedance rules during monitoring.

This construction is operationally reasonable for nursing preparedness. First, transparency: *T*_*t*_ and *S*_*t*_ define communicable baselines for staffing and stock planning, and residual spikes can be traced to specific months (23). Second, robustness: robust STL downweights anomalies during baseline fitting, reducing false positives from isolated outliers. Third, temporal portability: once windows and periods are fixed, the same pipeline applies prospectively to new months, ensuring consistent alert semantics aligned with the ranked disease set.

## 3 Results

### 3.1 Entropy-weighted pillars and disease profiles

**Figure 2:**
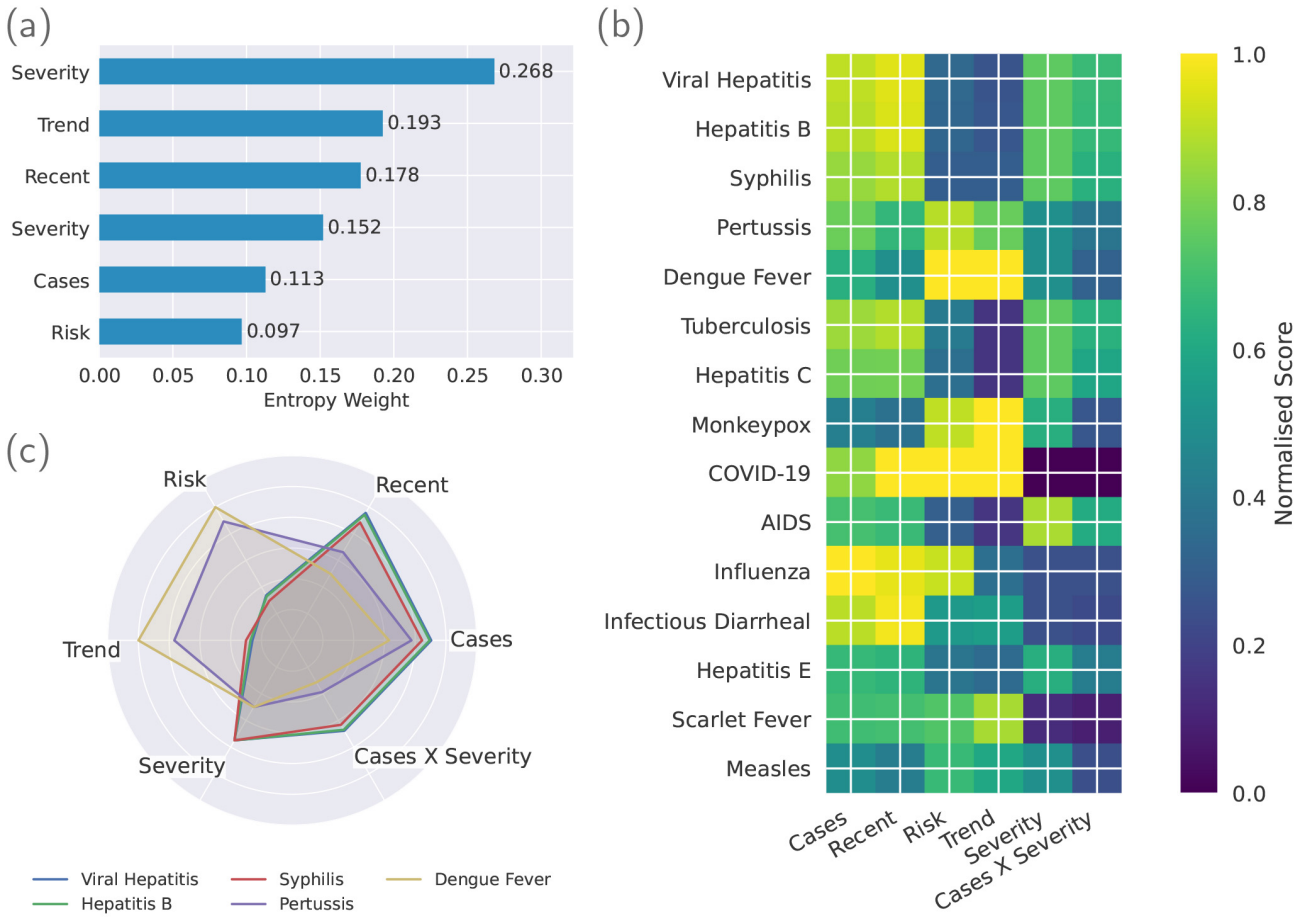
Pillar-weighting and disease-profile summaries. **(a)** shows the entropy-derived weights for the six pillars. **(b)** displays the normalized pillar matrix for the top-ranked diseases under the consensus objective. **(c)** shows radar profiles for a representative top-5 diseases, illustrating heterogeneity in pillar composition.

Figure 2 summarizes the properties of the consensus target before model training. Figure 2a shows the entropy weights produced by Equation 4, pillars exhibiting larger cross-disease dispersion are assigned larger weights, while uniformly uninformative pillars are de-emphasized. Figure 2b displays the normalized pillar matrix for the top 15 ranked diseases under the consensus objective (Equation 5), highlighting distinct mixes of *Burden, Recent, Risk, Trend, Severity*, and the interaction *Burden*×*Severity*. Figure 2c shows radar plots for a representative top 5 diseases, making the heterogeneity of pillar composition visually explicit: some diseases are driven by volume or recency, whereas others are prioritized because of volatility, trend, or high policy-informed severity (or their interaction).

These figures validate that the consensus target is (i) *data-adaptive*—the weighting reflects empirical discriminability across diseases; (ii) *interpretable*—each pillar has a clear operational meaning for nursing preparedness; and (iii) *discriminative*–different diseases rise to the top for different, transparent reasons rather than via a single dominant factor. This provides a principled teacher signal to the learner in Section 2.2.2 and guards against ad-hoc manual weighting.

### 3.2 Disease-level cross-validation performance of the ranking learner

To quantify how well the trained scorer generalizes to unseen disease profiles, we evaluated the model with LODO cross-validation as specified in Section 2.2.3. Table 1 reports the four primary metrics computed from out-of-fold predictions across all 47 diseases.

**Table 1:** LODO cross-validation performance of the LightGBM ranking model.

Folds	MAE	RMSE	Spearman ρ_*s*_	Kendall τ_*k*_	Top-15 overlap
47	0.0544	0.0735	0.8511	0.6836	12/15 (80.0%)

Table 1 indicates strong rank preservation under disease-level holdout. In particular, ρ_*s*_ = 0.8511 and τ_*k*_ = 0.6836 show that the ordering induced by out-of-fold predictions remains largely consistent with the entropy-weighted consensus target, which is the central requirement for downstream top-*K* prioritization. Simultaneously, MAE and RMSE remain moderate relative to the target scale in [0, 1], indicating that rank agreement is not achieved by trivial compression. At the operational top-list level, the overlap between the teacher top-15 and the LODO-predicted top-15 is 80.0%, which remains substantial under a strict leave-one-disease-out setting and supports practical robustness of prioritization.

Supplementary Table S4 lists the top-15 diseases with the smallest absolute LODO errors, and Supplementary Table S5 lists the top-10 diseases with the largest absolute LODO errors. Larger out-of-fold errors are concentrated in diseases with atypical or bursty profiles (e.g., dengue fever and monkeypox). This pattern likely reflects the combination of (i) heterogeneous trajectories within a 36-month window, (ii) feature components that preserve within-year shape information, and (iii) the stringency of disease-level LODO validation, which requires generalization to a held-out disease type. These cases represent an expected limitation of the validation setting and are consistent with the model's intended use as a ranking tool rather than a case-count forecasting model.

To evaluate the value of multi-dimensional learning, we compared the learned scorer with common single-indicator prioritization rules (Burden-only, Recent-only, Trend-only, Risk-only, and Severity-only) against the same teacher target. The one-pillar rankings showed lower top-15 agreement (5/15 to 11/15) and weaker rank concordance than the learned scorer (12/15, ρ_*s*_ = 0.8511), suggesting that single-indicator triage may overlook important multi-dimensional risk patterns. Detailed results are reported in Supplementary Material Section 4.

### 3.3 Model interpretability and consensus alignment

Because predictive robustness alone does not reveal what the model has learned, we then examine model interpretability and consensus alignment to determine whether the learned scorer captures clinically and operationally meaningful signals rather than spurious correlations.

**Figure 3:**
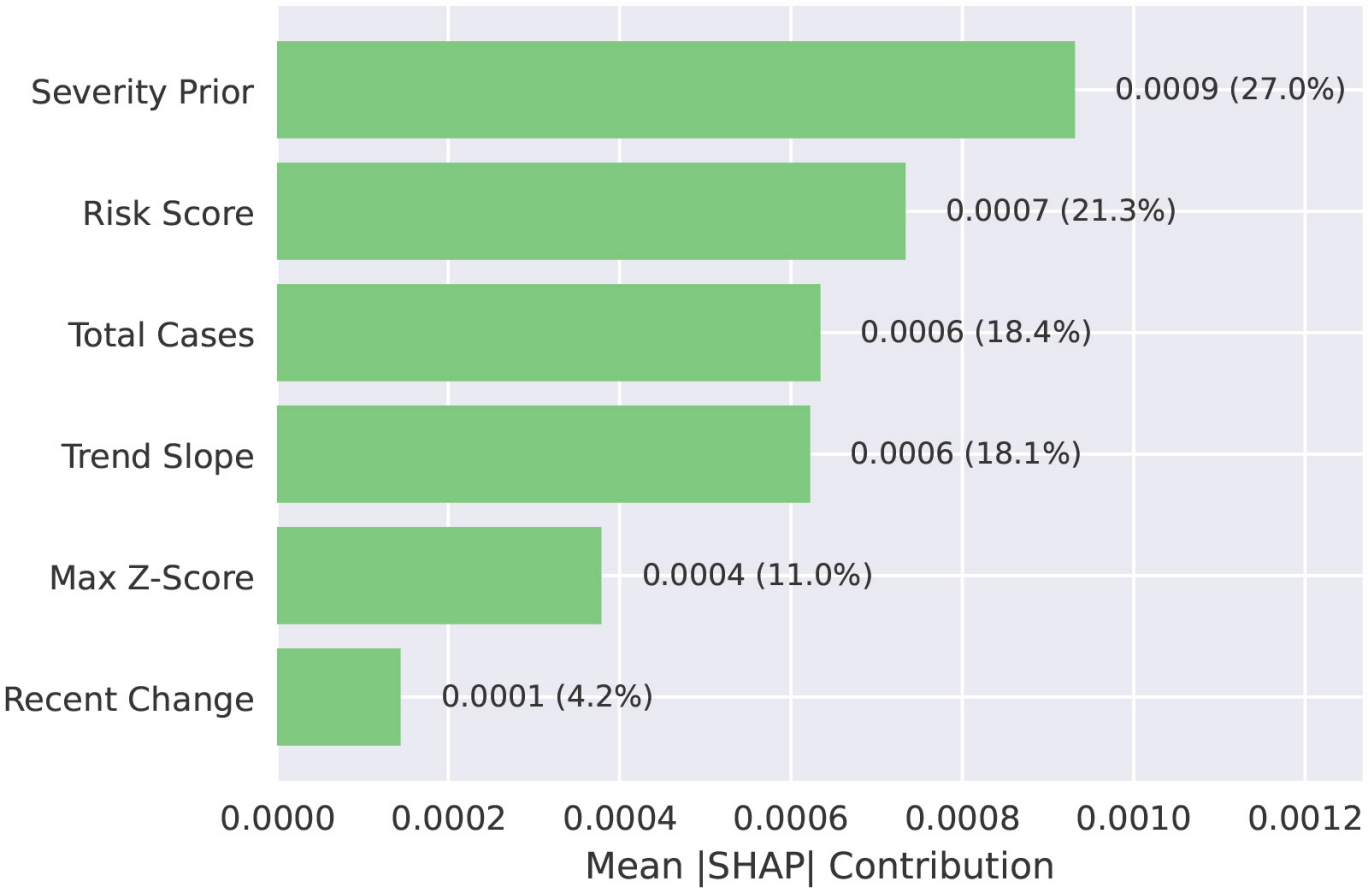
Feature importance via TreeSHAP. Mean absolute SHAP contributions for selected features in the trained LightGBM regressor. Bars quantify each feature's average magnitude of influence on the model score ŷ.

Figure 3 displays global feature importance derived from local TreeSHAP attributions. The percentage data in the figure are the results of normalizing all items. After training the LightGBM regressor, we computed exact per-sample, per-feature SHAP values on the training cohort. Then we aggregated them by taking the mean absolute contribution for each feature across diseases.^1^ This procedure shows how much, on average, each input feature in *X* (with dimensionality *p*≫6) shifts the model score ŷ away from the baseline, independent of sign, thereby providing a robust summary of influence.

The learned scorer places substantial weight on policy-informed Severity and on dynamical descriptors such as Risk (volatility/extremes), Total cases (burden), and Trend (direction of change), with smaller yet non-negligible influence from short-horizon change metrics. This qualitative concordance between entropy-derived priorities (Figure 2a) and *post-hoc* attributions (Figure 3) indicates that the model has learned a mapping *f*(*X*) that respects the same operational logic encoded in the consensus objective, rather than exploiting spurious artifacts.

The SHAP decomposition supplies two forms of validation for the proposed approach. First, SHAP's principal drivers align with the six pillars (e.g., severity, trend, volatility), validating the reliability of the teacher-student design (pillars as the teacher, model as the student). Second, SHAP decomposes each disease's score into explicit feature contributions and can be aggregated back to the pillar level, clearly explaining why a disease ranks highly (often: high severity + rising trend + elevated volatility) and supporting anticipatory staffing and supply preparation for nursing and infection control.

### 3.4 STL decomposition analysis

We provide empirical evidence that STL is suitable as the de-seasonalization step for alerting. All diagnostics are computed on the top-15 diseases selected by our ranking, using a 24-month calibration window to avoid look-ahead. We report the four measures defined in Equations 17–20 and interpret them jointly.

**Figure 4:**
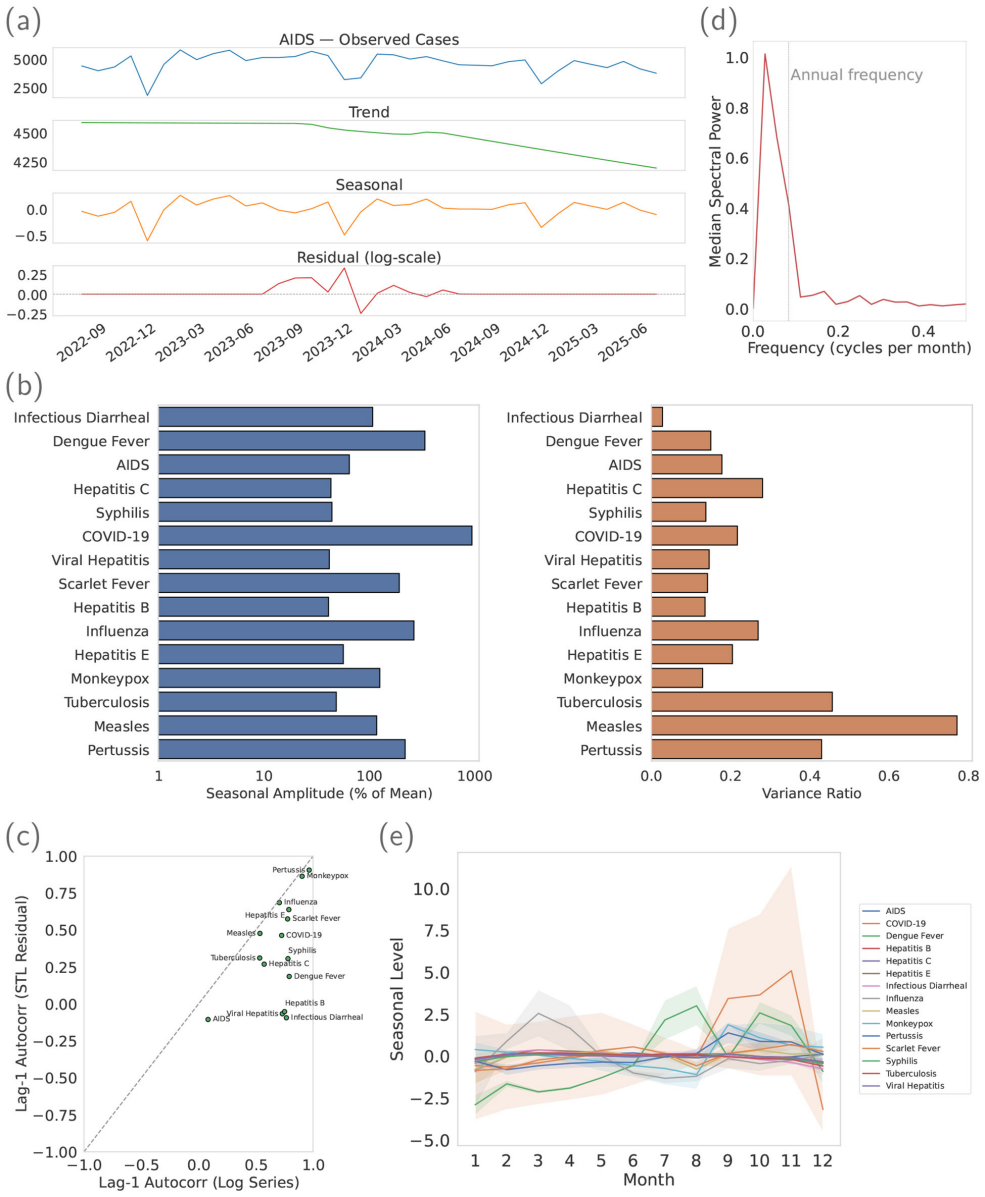
STL diagnostics overview. **(a)** shows the STL decomposition example (AIDS). **(b)** shows seasonality strength and variance reduction. **(c)** compares lag-1 autocorrelation before and after STL. **(d)** reports the median periodogram of log series across diseases. **(e)** summarizes the mean seasonal level by calendar month, and the shaded band indicates a 95% variability band (approximated from calibration-window monthly means).

Figure 4a illustrates an example for acquired immunodeficiency syndrome (AIDS), where STL separates the level, seasonality, and deviation components on the log scale. The seasonal panel provides a month-specific baseline that is easy to communicate for planning, while the residual panel concentrates unexpected departures from that baseline. In the calibration window, the residuals exhibit reduced variance and serial dependence relative to the raw series, supporting the use of simple, quantile-calibrated thresholding in the subsequent monitoring period.

Figure 4b shows that seasonal strength is consistently non-trivial across the analyzed diseases, while variance ratios are clearly below one. Taken together, these results indicate that STL removes a substantial and recurrent component of variability and yields residuals with reduced dispersion, which is advantageous for reliable alerts.

Figure 4c shows a systematic shift toward lower lag-1 autocorrelation after STL. Lower serial dependence means residuals are more amenable to simple, stable threshold rules, reducing the risk of chained false positives or negatives across adjacent months.

Figure 4d corroborates the presence of an annual cycle with a clear spectral peak near 1/12. Combined with the strong seasonal strength and improved residual diagnostics, this supports choosing *m* = 12 for decomposition in this dataset; the procedure remains data-driven and can accommodate other periods if indicated.

Figure 4e summarizes the seasonal templates by calendar month, with the shaded band showing an approximate 95% variability band for the monthly mean seasonal level, estimated from the calibration years. Bandwidth differs across diseases because it reflects disease-specific seasonal volatility and irregular outbreak perturbations; with a finite calibration window, these effects are more visible for diseases with unstable month-to-month seasonal shape. These baselines clarify what is normal for each month and make deviations in the residuals operationally interpretable.

The four diagnostics convey a consistent picture. Seasonal patterns are present and substantial, variance and autocorrelation are reduced after decomposition, and the dominant frequency is annual, justifying *m* = 12. The resulting residuals are therefore well suited to fixed-threshold alerting, while the explicit seasonal templates support clear communication of month-specific expectations to nursing and infection control teams.

### 3.5 Alerting analysis

#### 3.5.1 STL-residual alerting setup

**Figure 5:**
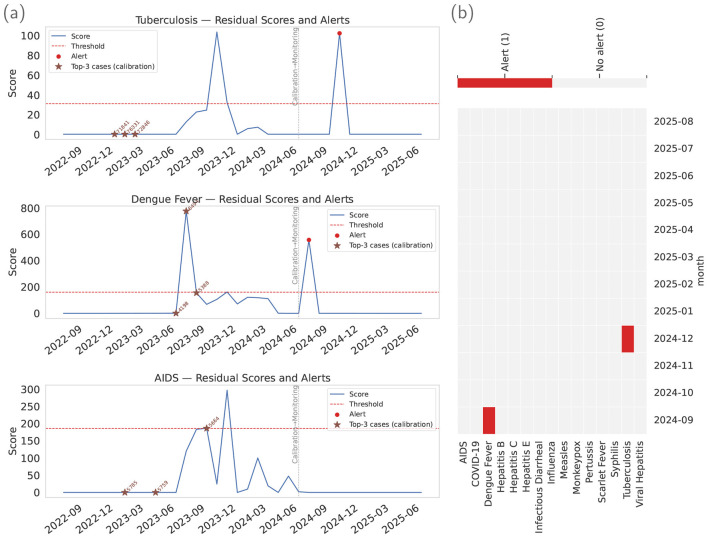
Alerting results overview. **(a)** stacks residual-score time series for representative diseases, showing the one-sided robust score, the quantile-calibrated threshold, and alert markers across the monitoring window. **(b)** summarizes portfolio alerts as a disease-by-month heatmap, highlighting temporally localized bursts and cross-disease co-occurrence.

For each disease, STL is fitted on the log scale, and the remainder ${\hat{R}}_{t}$ is converted to a one-sided, robust score ${\text{score}}_{t}=\max\left\{0,{\hat{R}}_{t}\right\}/\text{MAD}\left({\hat{R}}_{\text{calib}}\right)$ on a 24-month calibration window. We conducted alert evaluation experiments using the most recent 12 months of data, providing a fixed out-of-sample horizon that aligns with annual planning and reporting cycles in hospital nursing operations while remaining sufficiently long to capture seasonal variability. A disease-specific threshold τ(ρ) is then set as the (1−ρ) quantile of the calibration scores (with a small floor to prevent overly low cutoffs) and kept fixed in the subsequent monitoring period; an alert is issued when score_*t*_≥τ(ρ). This rule targets positive departures from the seasonal-trend baseline and yields comparable exceedance semantics across diseases; Figure 5a visualizes the resulting alert mechanics.

Figure 5a shows three representative diseases. Each panel plots the residual score over time, the disease-specific threshold (kept fixed within the monitoring window), alerts highlighted as red points, and the vertical line that separates the calibration window from the monitoring. Star markers indicate the top-3 months by raw cases within the calibration window. Two patterns are noteworthy: (i) alerts concentrate on months with positive departures from the seasonal-trend baseline rather than on routine seasonal peaks, and (ii) the same thresholding rule yields disease-specific alert timing because residual dynamics differ across diseases.

#### 3.5.2 Baseline comparisons against status-quo alerting

To address comparator requirements, we evaluated two operational baselines on the same 24-month calibration and 12-month monitoring split: (i) raw-count thresholding and (ii) a seasonal-naive residual rule (*x*_*t*_−*x*_*t*−12_), both using calibration quantile thresholds. In parallel, our method used the same quantile-controlled logic on STL residual scores. The objective is not exact peak-date forecasting, but selective exception reporting for atypical deviations with operationally usable alert burden.

**Figure 6:**
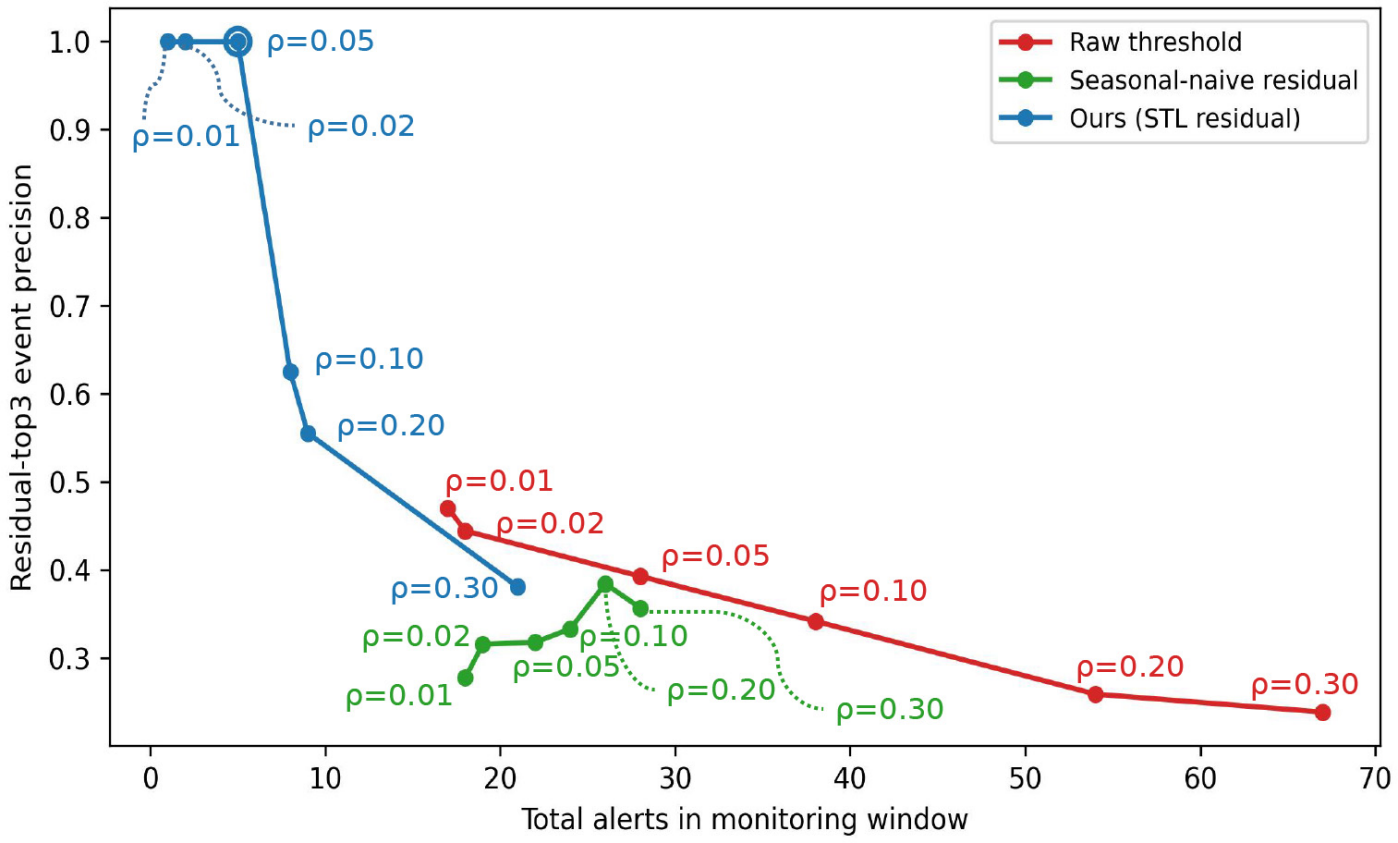
Stage-2 alerting trade-off (burden vs. residual-top3 event precision). We compare STL-residual alerting against two status-quo baselines (raw-count thresholding and a seasonal-naive residual rule) using the same 24-month calibration/12-month monitoring split. The x-axis is the total number of alerted disease months in monitoring (alert burden). The y-axis is residual-top3 event precision (micro-averaged): the fraction of alerted disease-months that coincide with disease-specific top-3 residual-extreme months in monitoring.

Figure 6 summarizes the burden—precision trade-off across alerting methods. The x-axis shows the total number of alerted disease months in the monitoring window (alert burden). The y-axis reports the *residual-top3 event precision* (micro-averaged), defined as the proportion of alerted disease-months that coincide with the disease-specific top-3 residual-extreme months in the monitoring window. This residual-top3 event precision is formalized in Equation 23. Formally, let *A*_*d*_ denote the set of alerted months for disease *d*, and let $R_{d}^{\left(3\right)}$ denote the set of the top-3 months ranked by residual anomaly magnitude for the same disease. The metric is defined as


$$\begin{array}{l} \text{Precisio}{\text{n}}_{\text{top}3}^{\text{res}}=\frac{\sum_{d}|A_{d}\cap R_{d}^{\left(3\right)}|}{\sum_{d}|A_{d}|}. \end{array}$$ (23)

A higher value indicates that the issued alerts are more concentrated on the most atypical residual months for each disease, rather than being broadly distributed across routine fluctuations. Because this metric specifically rewards concentration on residual extremes (rather than general peak-month capture), we interpret the comparator results as objective-dependent and use them to support operating-point selection for low-burden exception reporting, rather than to claim universal superiority of any single alerting rule.

Based on this trade-off, we selected a conservative operating point (ρ = 0.05, τ_min_ = 0) designed to maintain a low alert burden while preserving high specificity for exception reporting, thereby reducing alert fatigue in nursing workflows. Under the 24-month calibration / 12-month monitoring design, the STL-residual method generated 5 total alerts (mean of 0.333 alerts per monitored disease) and achieved a residual-top3 event precision of 100%. In comparison, raw-count thresholding generated 28 alerts (mean 1.867 per disease; residual-top3 event precision 39.3%), and the seasonal-naive residual-threshold rule generated 22 alerts (mean 1.467 per disease; residual-top3 event precision 31.8%). We report these results as an operating-point illustration under the chosen objective, not as a universal claim of superiority across all alert definitions.

For operational illustration, Figure 5b shows the resulting portfolio alert map under a more conservative deployment setting (ρ = 0.05, τ_min_ = 0.05). This differs from the comparator operating-point selection in Figure 6 (where τ_min_ = 0 was used to let ρ directly govern the burden–precision trade-off). Here, the positive minimum score floor τ_min_ suppresses low-magnitude fluctuations and yields a sparser alert pattern. In Figure 5b, rows correspond to diseases and columns to months, and marked cells indicate alert months. The resulting sparse pattern supports interpretable cross-disease coordination and action prioritization. Two signals (dengue in September 2024 and tuberculosis in December 2024) are presented as illustrative plausibility checks consistent with epidemiological context (48–51), rather than as stand-alone external validation.

Window-length sensitivity analyses were performed to address the practical concern that a 24-month history might be short for some seasonal diseases. In principle, longer histories are preferable because they provide more seasonal cycles and can improve the stability of seasonal templates and threshold calibration. Consistent with this expectation, our comparisons indicate that the 36-month design (24-month calibration/12-month monitoring) yields more stable and selective alerting behavior than a shorter 24-month design (12-month calibration/12-month monitoring), including better comparator stability. Detailed window-length comparison results are provided in Supplementary Material Section 5. Our method introduces a calibration and monitoring framework that can be applied to longer histories when available.

## 4 Discussion

This study addresses a practical gap in nursing-oriented surveillance: hospitals must allocate scarce staff and supplies under imperfect, seasonal, and sometimes delayed reporting. We propose a two-stage framework that links prioritization and alerting into a single auditable decision chain. Stage 1 aggregates six policy- and epidemiology-grounded pillars into an entropy-weighted consensus target and distills it into a scorer learned from routinely derived features, with local recalibration when deployed in new settings. Stage 2 applies STL-based residual monitoring to the top-*K* diseases and generates month-stamped alerts that emphasize atypical increases relative to seasonal–trend baselines rather than routine peaks.

Operationally, the intended loop is monthly: update the disease ranking from newly released surveillance data, monitor a tractable shortlist, issue residual alerts, trigger predefined actions (rota adjustment, personal protective equipment and consumable checks, cohorting and isolation preparation, and cross-team communication), and review alert burden at month-end. Compared with status quo single-indicator practice, this workflow provides both prioritization and selective alerting while retaining interpretability through pillar profiles/TreeSHAP and STL components.

The multi-indicator design is important because burden alone can over-privilege endemic high-volume conditions, whereas risk or trend alone can overreact to noise. Entropy weighting provides a data-adaptive alternative to manual weighting, and learning the scorer from richer features avoids fixing a single linear mixture. The STL layer complements ranking by separating expected seasonal–trend structure from deviations, enabling quantile-calibrated thresholds with clear exceedance semantics and tunable burden–sensitivity trade-offs.

Several limitations are present in this study. The consensus target depends on surveillance quality and a context-dependent severity prior; both ranking and alerting can be affected by under-reporting or coding shifts. STL assumes a reasonably stable seasonal template within the calibration horizon, and threshold calibration requires choices that trade sensitivity against workload, especially when monitoring many diseases. Our framework provides contemporaneous anomaly flagging rather than exact peak-time prediction, so its operational value is strongest for next-cycle preparedness. While the framework is methodologically portable, external validation on other regions/hospitals is still needed. Portability requires: (i) a disease-by-time count series at comparable temporal granularity; (ii) locally defined severity priors reflecting clinical/public-health consequences; and (iii) local recalibration of operating thresholds (e.g., ρ) to match alert burden and resources. Future work includes prospective implementation studies measuring workflow impact and false-alarm costs, and the incorporation of routinely available covariates to improve timeliness.

## 5 Conclusion

We proposed a two-stage framework that supports nursing preparedness under data and resource constraints: a multi-indicator ranking that consolidates policy and epidemiology-grounded pillars into a consensus target and a learned scorer, followed by STL-based residual monitoring with quantile-calibrated thresholds for operational alerts. The ranking component prioritizes diseases based on a transparent, data-adaptive synthesis of burden, recency, risk, trend, severity, and their interactions, while the alerting component focuses on atypical increases relative to seasonal-trend baselines rather than routine peaks.

Results indicated that seasonal patterns are substantial, residual variance and autocorrelation are reduced after decomposition, and alerts concentrate on meaningful deviations with clear exceedance semantics. The framework offers a pragmatic path from routine surveillance to proactive planning action: it tells teams which diseases matter most and when unusual increases occur, with explanations that clinicians and nursing managers can inspect and trust. In this study, temporal generalization is supported by out-of-sample monitoring over the most recent 12 months; cross-region deployment remains future work requiring local recalibration and external validation.

## Data Availability

Publicly available datasets were analyzed in this study. This data can be found here: The datasets analyzed for this study can be found in the National Disease Control and Prevention Administration of China: https://www.ndcpa.gov.cn/jbkzzx/c100016/common/list.html.

## Footnotes

1 Where applicable, we report the ranking of features using mean |ϕ_*ik*_|; this aggregation is standard for tree ensembles and yields a scale-comparable global importance profile.
