# Supplementary material for: PrepCare: a two-stage framework for infectious disease prioritization and alerts for nursing preparedness
Source: Front Public Health. 2026 Mar 27;14:1776109. doi: 10.3389/fpubh.2026.1776109 (PMC13066142; doi:10.3389/fpubh.2026.1776109)
Supplement: Supplementary file 1 [file Data_Sheet_1.pdf]

Supplementary Material

1 DISEASE SEVERITY RISK PRIOR SCORES

Table S1 lists the disease severity prior scores used in the ranking framework. Note that higher severity (e.g., Class A/B) reflects consequence, not necessarily higher time-series volatility as measured by Risk.

Table S1: Disease severity prior scores.

Disease	Severity score
Plague	1.00
Cholera	1.00
Rabies	0.95
Human infection with highly pathogenic avian influenza	0.95
Severe acute respiratory syndrome (SARS)	0.95
AIDS	0.95
COVID-19	0.95
Viral hepatitis	0.90
Syphilis	0.90
Hepatitis B	0.90
Hepatitis C	0.90
Hepatitis D	0.90
Tuberculosis	0.90
Epidemic hemorrhagic fever	0.90
Human infection with avian influenza	0.90
Malaria	0.90
Hepatitis E	0.85
Unclassified hepatitis	0.85
Poliomyelitis	0.85
Diphtheria	0.85
Neonatal tetanus	0.85
Anthrax	0.85
Epidemic meningococcal meningitis	0.85
Monkeypox	0.85
Dengue fever	0.80
Measles	0.80
Pertussis	0.80
Typhoid and paratyphoid fever	0.80
Bacterial and amoebic dysentery	0.80
Japanese encephalitis	0.80
Hepatitis A	0.80
Typhus	0.80
Leptospirosis	0.75

Disease	Severity score
Schistosomiasis	0.75
Brucellosis	0.75
Gonorrhea	0.75
Visceral leishmaniasis	0.75
Filariasis	0.70
Echinococcosis	0.70
Influenza	0.70
Other infectious diarrhea	0.70
Acute hemorrhagic conjunctivitis	0.65
Scarlet fever	0.65
Mumps	0.65
Hand, foot, and mouth disease	0.60
Rubella	0.60
Leprosy	0.60

2 HYPERPARAMETER CANDIDATES AND SELECTION

We prespecified eight LightGBM hyperparameter candidate configurations and evaluated them using disease-level leave-one-disease-out (LODO) cross-validation. To reduce tuning variance under the small disease-level sample size, the candidate set was defined as a compact prespecified set rather than a full Cartesian grid. The tested configurations spanned key modeling dimensions, including tree complexity (max leaves per tree), learning rate, ensemble size, row and feature subsampling fraction, and minimum leaf sample size, with a fixed random seed (42) for reproducibility in Table S2. Candidates were ranked using the prespecified deterministic selection rule described in the main manuscript (primary criterion: top-15 overlap; tie-breakers: Spearman rank correlation, then MAE). The selected configuration is identified in Table S3.

Table S2: Prespecified LightGBM hyperparameter candidates.

Candidate	Max leaves	Learning rate	Ensemble size	Row subsampling	Feature subsampling	Minimum leaf sample size
1	31	0.05	500	0.9	0.8	6
2	31	0.03	800	0.9	0.8	6
3	31	0.10	300	0.9	0.8	6
4	15	0.05	500	0.9	0.8	6
5	63	0.05	500	0.9	0.8	10
6	31	0.05	500	0.7	0.7	6
7	31	0.05	500	1.0	1.0	6
8	31	0.05	700	0.9	0.8	3

Table S3: Candidate-wise leave-one-disease-out (LODO) validation performance used for hyperparameter selection.

Candidate	MAE	RMSE	Spearman ρ_s	Kendall τ_k	Overlap@15	Selected
6	0.0544	0.0735	0.8511	0.6836	0.8000	Yes
7	0.0541	0.0755	0.8505	0.6762	0.8000	No
2	0.0569	0.0770	0.8488	0.6762	0.8000	No
1	0.0567	0.0772	0.8422	0.6707	0.8000	No
4	0.0567	0.0772	0.8422	0.6707	0.8000	No
3	0.0609	0.0795	0.8175	0.6411	0.8000	No
5	0.0546	0.0740	0.8523	0.6855	0.7333	No
8	0.0664	0.0850	0.8070	0.6392	0.7333	No

3 MODEL VALIDATION OUTPUTS

Table S4 and Table S5 summarize disease-level leave-one-disease-out (LODO) out-of-fold prediction errors for our ranking model.

Table S4: Diseases with the 15 smallest absolute errors in leave-one-disease-out (LODO) out-of-fold predictions.

Disease (source label)	Final target	LODO pred	Abs. error	Target rank	Pred rank
Hepatitis B	0.6464	0.6460	0.0004	2	1
Anthrax	0.3995	0.4006	0.0011	24	20
Leptospirosis	0.3493	0.3479	0.0014	29	27
Echinococcosis	0.3518	0.3548	0.0030	28	25
Schistosomiasis	0.2264	0.2223	0.0041	40	43
Tuberculosis	0.6076	0.6026	0.0051	6	3
Bacterial and amoebic dysentery	0.4392	0.4469	0.0077	18	17
Diphtheria	0.2334	0.2229	0.0105	39	42
Human infection with avian influenza	0.1478	0.1371	0.0108	45	46
Other infectious diarrhea	0.5334	0.5192	0.0141	12	8
Scarlet fever	0.4827	0.4676	0.0151	14	13
Human infection with highly pathogenic avian influenza	0.2497	0.2341	0.0156	38	41
Japanese encephalitis	0.2830	0.2998	0.0168	36	36
Poliomyelitis	0.1288	0.1478	0.0190	46	44
Viral hepatitis	0.6504	0.6294	0.0211	1	2

Table S5: Diseases with the 10 largest absolute errors in leave-one-disease-out (LODO) out-of-fold predictions.

Disease (source label)	Final target	LODO pred	Abs. error	Target rank	Pred rank
Dengue fever	0.6096	0.3801	0.2295	5	23
Monkeypox	0.5627	0.3892	0.1735	8	22
Epidemic hemorrhagic fever	0.4447	0.2995	0.1452	16	37
Hepatitis A	0.3203	0.4634	0.1430	32	14
Acute hemorrhagic conjunctivitis	0.3357	0.4513	0.1156	30	16
Rubella	0.1556	0.2704	0.1148	44	39
Leprosy	0.1858	0.3004	0.1146	42	35
Typhus	0.2147	0.3222	0.1074	41	31
Mumps	0.3259	0.4256	0.0997	31	18
COVID-19	0.5608	0.4754	0.0855	9	12

4 STAGE-1 RANKING COMPARATOR RESULTS

To benchmark the Stage-1 ranking learner against simple status-quo prioritization rules, we compared single-pillar baselines with the same entropy-weighted consensus target used in the main analysis.

Table S6: Performance of Stage-1 single-pillar ranking baselines against the entropy-weighted consensus target.

Methods	MAE	RMSE	Spearman ρ_s	Kendall τ_k	Top-15 overlap
LightGBM (Ours)	0.0544	0.0735	0.8511	0.6836	12/15 (80.0%)
Recent-only	0.2013	0.2330	0.7689	0.5785	11/15 (73.3%)
Burden-only	0.1901	0.2213	0.7714	0.5872	10/15 (66.7%)
Trend-only	0.1989	0.2414	0.4817	0.3358	9/15 (60.0%)
Severity-only	0.2618	0.3196	0.2147	0.1637	7/15 (46.7%)
Risk-only	0.2802	0.3437	0.0445	0.0158	5/15 (33.3%)

5 STAGE-2 ALERT COMPARATOR RESULTS

Compared with the 24-month calibration / 12-month monitoring design, the shorter 12-month calibration / 12-month monitoring design exhibits markedly reduced calibration stability, most notably for the seasonal-naive baseline, which produces a substantially higher alert burden. Taken together, these results support the use of a 36-month history as a practical minimum for stable operation under the currently available data.

Table S7: Window-length sensitivity comparison of Stage-2 alerting (36-month vs 24-month design).

Setting	Method	Total alerts (monitor)	Mean alerts per disease	Residual-top3 event precision
24+12 (36-month window)	Ours	5	0.333	100%
24+12 (36-month window)	Raw threshold	28	1.867	39.3%
24+12 (36-month window)	Seasonal-naive residual	22	1.467	31.8%
12+12 (24-month window)	Ours	22	1.467	72.7%
12+12 (24-month window)	Raw threshold	24	1.600	16.7%
12+12 (24-month window)	Seasonal-naive residual	180	12.000	25.0%
